# Ex vivo comparison of sliding knot ligatures vs. haemostatic clips for equine small intestinal mesenteric vessel occlusion

**DOI:** 10.1186/s12917-020-02498-x

**Published:** 2020-08-12

**Authors:** Gessica Giusto, Marco Gandini

**Affiliations:** Department of Veterinary Sciences, University of Turin, Largo Braccini 2-5, 10095 Grugliasco, TO Italy

**Keywords:** Horse, Colic, Surgery, Haemostasis, Anastomosis

## Abstract

**Background:**

In equine abdominal surgery, resection and anastomosis of strangulated intestine is a commonly performed procedure. To date, ligatures, vessel sealing devices and the ligate-divide stapler have been described for this use in horses. The objective of this study was to compare the application of haemostatic clips and ligatures to occlude equine mesenteric vessels.

Portions of jejunum with ten associated mesenteric vessels were collected from 12 horses at a local abattoir and divided into two groups. Portions of intestine were divided into two sections comprising five vessels each and assigned to Group A or Group B. Each vessel was occluded with a triple ligature. In Group A, vessels were ligated with three circumferential ligatures tied with a sliding knot with two overthrows. In Group B, vessels were occluded with application of three haemoclips. The procedures were performed by the same experienced surgeon. Intestinal length, construction time and vessel leaking pressure were measured and compared between groups.

**Results:**

The intestinal length (mean ± SD) was 3.78 ± 0.43 m in Group A and 3.04 ± 0.83 m in Group B. The difference was not significant (*p* = 0.297).

The construction time (mean ± SD) was 7.03 ± 0.34 min in Group A and 2.40 ± 0.43 min in Group B. The difference was significant (*p* < 0.0001).

The leaking pressure was 1000 (750–1050) mmHg (median, IQ range) in Group A and 1050 (800–1050) mmHg (median, IQ range) in Group B. The difference was not significant (*p* = 0.225).

**Conclusions:**

Haemoclip application is comparable in terms of leaking pressure but quicker than sliding knots to apply.

## Background

In equine abdominal surgery, resection and anastomosis is a commonly performed procedure. Many horses with small intestinal disease have strangulating lesions that may often require resection [1].

Extensive small intestinal resection and anastomosis may require ligating and transecting of many vessels, necessitating considerable surgical time [1–3].

To provide haemostasis, single or double absorbable ligatures, surgical staplers and vessel sealing devices have been proposed and tested in horses [4–6].

For suture ligation, both monofilament and multifilament sutures can be used, but monofilament sutures were more resistant to fluid pressure in an in vitro study [4]. While many knots can be used for this purpose, a sliding knot resulted in comparable resistance to pressure to a surgeon’s knot but was quicker to perform [3]. In fact, Gandini et al. (2014) [4] described the use of a sliding knot to provide effective haemostasis in mesenteric vessels while reducing surgical time compared to other haemostatic ligatures.

In recent years, to reduce surgical time, vessel sealing devices have been employed in horses for this purpose, although they cannot always be used when the mesentery is oedematous or with large vessels [2, 3, 5], having the ability of occluding vessels only up to 7 mm in diameter. In a study comparing vascular occlusion with double suture ligation, surgical staplers and an electrosurgical vessel sealing device, the bursting pressure was greater than systolic pressure in conscious or anaesthetised horses for all three methods and the suture ligation was strongest, followed by electrosurgical vessel sealing [3].

For resection of longer sections of small intestine, vessel ligation using staples or mechanical devices is much faster than suture ligation. The benefit of the surgical time gained can outweigh the additional costs associated with vessel sealing devices [3].

Additionally, the use of ligate-divide staplers has been reported in horses [3] but these devices appear to be difficult to find and not commercially available at the time of writing.

High cost is also a limitation in the use of staplers or sealing devices, although the latter, despite being sold for single-patient use, can be safely re-sterilised [7–9].

Manual application of single metallic clips has been described in human surgery and cited in animals as well, although, in horses, their use is only anecdotally reported [10].

Vascular clips have been widely used in human surgery and have been demonstrated to be safe and effective in urogenital or gynaecological procedures [11, 12]. In several studies, the use of vascular clip compared favourably with ligatures and vessel sealing devices [13, 14]; thus, they can be a valid alternative to vessel sealing devices.

To date, no study has evaluated the application of single metallic clips on mesenteric vessels in horses.

Our hypothesis was that haemoclips would be as effective as ligatures in occluding equine mesenteric vessels in resisting comparable hydrostatic pressures while being quicker to apply.

The purpose of this study was to compare two methods, haemoclips and ligatures, for providing haemostasis during small intestinal resection and anastomosis in horses.

## Results

### Intestinal length

The unpaired T test was used to compare bowel length of specimens. The bowel length of specimens in Group A was 3.78 ± 0.43 m (mean ± SD) and 3.04 ± 0.83 m (mean ± SD) in Group B. The difference was not significantly different between groups (*p* = 0.297).

### Construction time

The construction time was compared with the unpaired T test. Construction time was 7.03 ± 0.34 min (mean ± SD) in Group A and 2.40 ± 0.43 min (mean ± SD) in Group B. The difference was highly significantly different between groups (*p* < 0.0001).

### Leaking pressure

There was no leakage or rupture from the arteries during pressure testing. All the pressure tests produced a leak of coloured fluid on the side of the artery opposite the catheter insertion site.

Pressure data were not normally distributed; thus, the leaking pressures were compared with the Mann–Whitney test. The leaking pressure was 1000 (750–1050) mmHg (median, IQ range) in Group A and 1050 (800–1050) mmHg (median, IQ range) in Group B.

The difference was not significantly different between groups (*p* = 0.225).

## Discussion

This study compared the use of sliding knots with haemostatic clip application for mesenteric vessel closure in small intestinal resection in horses. Haemoclips were as effective as ligatures in occluding mesenteric vessels but quicker to apply.

Surgery time is an important factor in equine abdominal surgery [1–6]. Extensive intestinal resection, when a considerable length of intestine is removed, is time consuming mostly because of the time needed for vessel closure. For this reason, some surgeons prefer to use stapling and ligating devices with the addition of a ligature for better haemostatic efficacy [2] or vessel sealing devices (LigaSure) [5]. Nevertheless these devices are expensive or have limitations to their use in large vessels or with oedematous or thickened tissues.

Only staples and ligatures transfix the vessels, thus slippage of the clip can occur.

Tobias [15] reported that a manual clip can be applied quickly and accurately to seal mesenteric vessels but Monnet and Orton [16] reported that they are more easily dislodged than suture ligatures. Since those reviews, haemoclips have evolved both in design and materials and new designs of clip have considerably reduced the risk of slippage. The haemoclips used in our study had a heart-shaped cross-section designed to give each clip a firm grip on the vessels (manufacturer description), which likely prevents slippage during intestine handling, although the possibility of leakage must always be considered by the surgeons despite the haemostatic system used. The risk of slippage can be avoided by using the correct size of clip relative to the vessels being occluded, applying the clips at a 90° angle with the vessels and leaving at least 1 mm of tissue distal to the most distal clip [17]. The application of two clips will further reduce the risk of postoperative leakage [17].

The haemoclips available were of three different sizes (small, medium and large) and allowed occlusion of vessels with a diameter between 2 and 16 mm. Therefore, in equine surgery, the large size of the manual haemoclips are proper and effective in size for use in vessels with oedematous or thickened tissue.

Ligatures may provide a more effective means of haemostasis in such cases but are time consuming, although sliding knots with a monofilament suture were quicker but as effective in providing haemostasis compared to other ligatures [4].

In this study, haemoclips were significantly quicker than sliding knots in closing mesenteric vessels. Although saving a few minutes may not seem clinically significant in a surgery lasting one to three hours, it could nevertheless contribute in reducing total surgical time.

Every little step in this direction may improve outcome, as surgical time is inversely correlated with survival in equine colic surgery.

Leaking pressures for both ligatures and clips were well above the physiological values of systolic pressure in horses, thus, likely to be effective in a clinical setting.

The limitations of our study include the fact that the operator that performed the vessel occlusion was obviously not blinded, and this could lead to potential bias in the construction time.

Our study was limited to ex vivo testing, and we cannot rule out the possibility that ligatures could behave differently when applied to live animals in pathological conditions. In fact, is well known that oedematous or weak tissue found in pathological conditions (e.g. mesenteric haematoma or oedema following strangulation) behave differently to the healthy tissue used in this study. Surgeons must be aware of the limitation common to all devices except for sutures.,

Limitations are given by the presence of oedematous tissue that can impair the effectiveness of clips, staples or thermal devices but have no or little effect on well placed sutures.

Nevertheless, proper elimination of the surrounding mesentery around vessels before application is paramount despite the occlusion method used, in order to reduce the risk of leakage. Further, in a clinical setting, haemoclip size may not adequate in all cases and their application can be dependent on vessel size.

A large, blinded, clinical study comparing the different hemostasis methods might be valuable to determine the optimum hemostasis methods in equine colic surgery.

This study can represent a starting point for possible future investigations. Clinical trials comparing ligatures with other means of achieving haemostasis in mesenteric vessels would be useful to formulate guidelines for colic surgery.

## Conclusions

We can conclude that when ligating mesenteric vessels in horses, haemostatic clips are a valid alternative, as in human surgery, to ligatures closed with sliding knots in providing vessel occlusion based on bursting pressure but are quicker to apply.

## Methods

Twelve portions of jejunum starting 1 m distal to the duodenocolic ligament, complete with mesentery and mesenteric vessels, were harvested soon after death from 12 healthy slaughtered horses (mean age 26 months, range 18–30 months, mean weight 450 kg, range 420–480) at the Didactical Abattoir, Department of Veterinary Sciences, University of Turin, and were washed, cleaned and stored in warm 0.9% sodium chloride solution. The experiments were performed within six hours following collection. Each specimen included a length of intestine with ten associated mesenteric arteries.

Specimens were divided in half and each portion, comprising five mesenteric vessels, assigned to Group A or Group B. In Group A, each mesenteric artery was ligated with three circumferential ligatures tied with a sliding knot with two overthrows [4] with a USP 0 suture.^1^

To avoid operator influence, all knots were performed by the same experienced surgeon. For each work session, four specimens were tested, to avoid surgeon fatigue. To mimic the clinical setting, vessels were dissected from the mesentery and the surgeon was aided by an assistant. After completion, the excess suture thread of each knot was cut to a standard length of 3 mm. The tensile force applied by the surgeon for creating the knot was not measured.

In Group B, each vessel was ligated with the application of three haemoclips^2^ in the same orientation. To mimic a clinical setting, an assistant held the cartridge with the clips and assisted the surgeon in loading each clip. Haemoclips come in three sizes from small to large. For this study ‘large’ haemoclips (7 mm) were used as per the manufacturer’s recommendation for the supposed vessels size (vessels should be between 1/3 and 2/3 of the clip size).

### Intestinal length

To ensure homogeneity between groups, the length of each specimen was measured.

For each specimen, the length in centimetres was measured on the antimesenteric border of the intestine and compared between groups.

### Construction time

Construction time was recorded and compared between groups; time was measured starting when the needle was inserted through the mesentery to perform the first knot and was stopped after the assistant cut the last knot’s thread.

For haemoclips, time started when the surgeon held the haemoclip applicator for the first time and was stopped when the last clip was in place. The time for freeing the vessels from the mesentery was not considered for both ligating techniques.

### Leaking pressure

After performing each ligature or haemoclip application, each mesenteric artery was transected at 3 mm distal from the second ligation (in a proximo-distal order). The artery was cannulated proximally about 5 mm from the proximal knot.

A 22G 1–1/4″ intravenous catheter was connected through a three-way stopcock to an analogue pressure gauge and a 50 ml syringe. The intravenous catheter was partially fed along the trocar to avoid puncture of the vessel and introduced into the artery. To avoid trauma to the vessels, two pieces of 5 mm latex tubing were placed around the jaws of a mosquito forceps. The artery was clamped around the catheter with the mosquito forceps. A solution of methylene blue dye and water was injected through the catheter until ligature or haemoclip failure, as identified by a loss in pressure or dye leakage [4].

An operator, blinded to the sample tested, performed the reading of maximum pressure sustained by the system. When reaching the value of 1050 mmHg, the test was terminated.

### Statistical analysis

The study power and sample size were calculated with a custom-made excel calculator with alpha level set at 0,05 and 90% power using the construction time.

Normal distribution of data was evaluated with the Kolmogorov–Smirnov test.

Since data of intestinal length and construction time were normally distributed, an unpaired T.

test was used. Leaking pressure data were not normally distributed and the Mann–Whitney test was used. All statistical analyses were performed using statistical software^3^ with significance set to *p* < 0.05.

## Data Availability

The datasets used and/or analysed during the current study are available from the corresponding author on reasonable request.

## Footnotes

1 Ethicon PDS II, Johnson & amp; Johnson Medical Spa, Via del Mare 56, 00071, Pomezia, Rome, Italy

2 Hemoclip Weck, Teleflex Medical, 2917 Weck Drive, Research Triangle Park, NC 27709 USA

3 Prism 8, GraphPad software, 2365 Northside Drive, Suite 560 San Diego, CA 92108
